# Real-Time 2D MR Cine From Beam Eye’s View With Tumor-Volume Projection to Ensure Beam-to-Tumor Conformality for MR-Guided Radiotherapy of Lung Cancer

**DOI:** 10.3389/fonc.2022.898771

**Published:** 2022-06-29

**Authors:** Xingyu Nie, Guang Li

**Affiliations:** ^1^ Department of Medical Physics, Memorial Sloan Kettering Cancer Center, New York, NY, United States; ^2^ Department of Radiology, University of Kentucky, Lexington, KY, United States

**Keywords:** MR-BEV-cine-guided radiotherapy, beam-to-tumor conformality, real-time motion prediction, latency, Motion management

## Abstract

**Purpose:**

To minimize computation latency using a predictive strategy to retrieve and project tumor volume onto 2D MR beam eye’s view (BEV) cine from time-resolved four-dimensional magnetic resonance imaging (TR-4DMRI) libraries (inhalation/exhalation) for personalized MR-guided intensity-modulated radiotherapy (IMRT) or volumetric-modulated arc therapy (VMAT).

**Methods:**

Two time-series forecasting algorithms, autoregressive (AR) modeling and deep-learning-based long short-term memory (LSTM), were applied to predict the diaphragm position in the next 2D BEV cine to identify a motion-matched and hysteresis-accounted image to retrieve the tumor volume from the inhalation/exhalation TR-4DMRI libraries. Three 40-s TR-4DMRI (2 Hz, 3 × 80 images) per patient of eight lung cancer patients were used to create patient-specific inhalation/exhalation 4DMRI libraries, extract diaphragmatic waveforms, and interpolate them to *f =* 4 and 8 Hz to match 2D cine frame rates. Along a (40•*f*)-timepoint waveform, 30•*f* training timepoints were moved forward to produce 3×(10•*f*-1) predictions. The accuracy of position prediction was assessed against the waveform ground truth. The accuracy of tumor volume projections was evaluated using the center-of-mass difference (∆COM) and Dice similarity index against the TR-4DMRI ground truth for both IMRT (six beam angles, 30° interval) and VMAT (240/480 beam angles, 1.5°/0.75° interval, at 4/8 Hz, respectively).

**Results:**

The accuracy of the first-timepoint prediction is 0.36 ± 0.10 mm (AR) and 0.62 ± 0.21 mm (LSTM) at 4 Hz and 0.06 ± 0.02 mm (AR) and 0.18 ± 0.06 mm (LSTM) at 8 Hz. A 10%–20% random error in prediction-library matching increases the overall uncertainty slightly. For both IMRT and VMAT, the accuracy of projected tumor volume contours on 2D BEV cine is ∆COM = 0.39 ± 0.13 mm and DICE = 0.97 ± 0.02 at 4 Hz and ∆COM = 0.10 ± 0.04 mm and DICE = 1.00 ± 0.00 at 8Hz.

**Conclusion:**

This study demonstrates the feasibility of accurately predicting respiratory motion during 2D BEV cine imaging, identifying a motion-matched and hysteresis-accounted tumor volume, and projecting tumor volume contour on 2D BEV cine for real-time assessment of beam-to-tumor conformality, promising for optimal personalized MR-guided radiotherapy.

## Introduction

One of the major advantages of magnetic resonance imaging integrated linear accelerator (MR-Linac) is to provide MR-guided radiotherapy (MRgRT), including real-time tumor motion management, making respiratory gating accurate and tumor tracking possible, especially for hypo-fractional stereotactic body radiotherapy (SBRT) (1–3). As MRgRT provides patient-specific imaging in real-time during treatment, adapting to inter-fractional and intra-fractional patient anatomic variations, it offers the best-personalized radiotherapy. Clinically, improved treatment outcomes have been reported showing the benefit of sparing critical organs at risk (OARs) so that the tumor lethal dose can be prescribed and delivered to a mobile target, including lung, liver, and pancreatic cancer (3, 4). Compared with conventional image-guided radiotherapy (IGRT), MRgRT offers many advantages, including real-time imaging with high soft-tissue contrast without ionization radiation. So far, the intensity-modulated radiotherapy (IMRT) technique is available in the MR-Linac and the volumetrically modulated arc therapy (VMAT) technique should be possible in the future.

Currently, dynamic 2D cine imaging in the sagittal and coronal views can be employed for MR-guided IMRT to monitor respiratory-induced tumor motion in real time covering the major motions in the superior–inferior (SI) and anterior–posterior (AP) directions. Although the two cine views infer a 3D tumor motion, they are partial and indirect views of a volumetric tumor related to the radiation beam. Moreover, potential through-plane tumor motion may interfere with motion interpretation. The more effective, optimal view for assessing the beam-to-tumor conformality should be the beam eye’s view (BEV), which is how the radiation beam sees the mobile tumor and only needs one cine scan (5–7). Previously, a 2D BEV cine technique with tumor volume projection has been reported feasible for better MRgRT (8). For IMRT treatment, adequate accuracy and performance have been achieved to identify and project a volumetric tumor onto the BEV by 2D–3D matching between the 2D tumor image on the BEV cine images and a time-resolved (TR) 4DMRI library containing volumetric images from multiple breathing cycles (9–12). For MR-guided VMAT, which may be available in the future, real-time communication and computation are required for 2D cine imaging with a rotating BEV and projecting tumor volume with minimal latency.

To overcome system latency, predictive strategies have been applied to provide a just-in-time prediction of tumor motion in the next 30–1,000 ms, using conventional Linac for tumor tracking, including adjusting the radiation beam, a multi-leaf collimator (MLC), or couch position to keep up with a tumor motion (13–15). Various predictive methods have been evaluated, including time-series-based (16), model-based (17), regression-based (18), and machine-learning-based (19, 20) prediction methods. Short-term motion prediction is an effective and efficient approach to overcome the system latency and reduce the frequency of x-ray imaging.

Additionally, respiratory motion hysteresis is a commonly occurring phenomenon in patients, resulting in variations in tumor motion trajectory, orientation, and shape between inhalation and exhalation (21, 22). Therefore, even at the same tumor displacement in the superior–inferior (SI) direction, the tumor shape, orientation, and anterior–posterior (AP) and left–right (RL) positions may vary due to the motion hysteresis (15, 21–24). Therefore, without differentiating the inhalation and exhalation processes, it may add uncertainties in retrieving the motion-matched tumor volume for BEV projection.

In this simulation study, we aimed to minimize the latency in matching a tumor volume by predicting the respiratory motion to identify and project tumor volume in parallel with the next 2D BEV cine acquisition. In addition, to minimize the motion hysteresis effect, two TR-4DMRI image libraries of exhalation and inhalation were created by grouping volumetric images based on their moving directions. Two predictive algorithms, a conventional autoregression (AR) modeling and a deep-learning-based long short-term memory (LSTM) neural network, were applied and evaluated with the known waveforms. The accuracy of tumor volume projection on the 2D BEV cine images was evaluated against the ground truth embedded in the TR-4DMRI datasets. This improved 2D BEV cine technique was evaluated for both MR-guided IMRT and VMAT treatments.

## Methods

**Figure 1:**
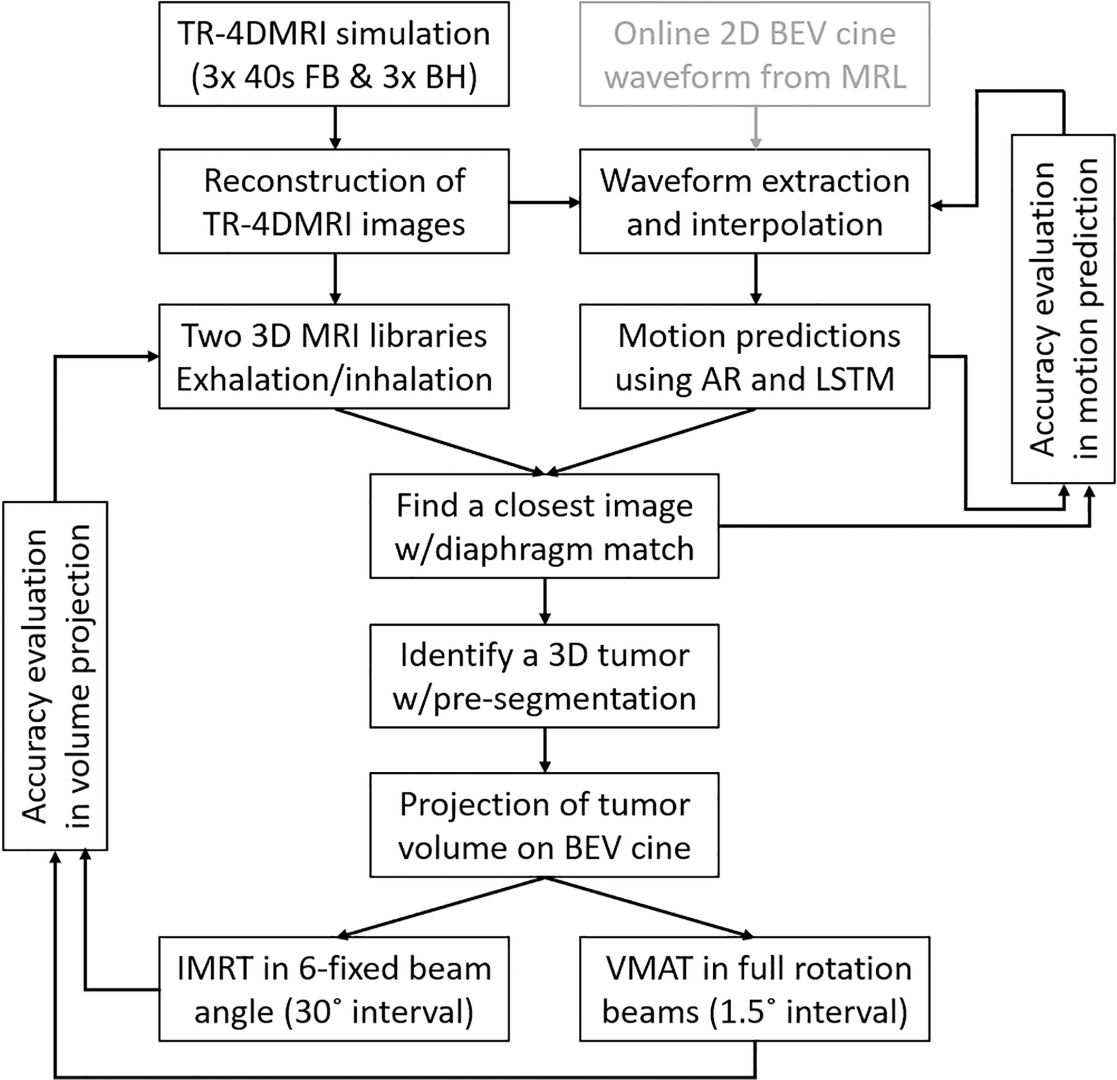
The workflow of the predictive strategy to predict, identify, and project tumor volume onto 2D BEV cine, followed by the verification of projected tumor volume using the center of mass and Dice similarity index against the ground truth for MR-guided IMRT and VMAT. In future clinical applications, the cine waveform can be utilized as well (gray box).

In this study, a predictive strategy was proposed and evaluated to identify and project tumor volume onto 2D BEV cine images in real time with minimized system latency and included respiratory motion hysteresis. The workflow of the strategy is shown in **Figure 1**.

### Three Time-Resolved 4DMRI Image Datasets, Waveform Extraction, and Interpolation

Eight lung cancer patients were recruited to participate in an IRB-approved protocol study using TR-4DMRI for respirator-induced tumor motion simulation and assessment in a 3T MRI scanner (Ingenia, Philips Healthcare, Amsterdam, Netherlands). Eight patients were scanned at simulation with 3D cine in free breathing (FB) for 40 (s) at *f =* 2* Hz* three times within one imaging session. A T1-weighted, multi-shot, turbo field echo pulse sequence was used with SENSE acceleration (6.0) and partial Fourier approximation (0.8), so a total of 3 × 80 volumetric 3D cine images with a voxel size of 5 × 5 × 5 mm^3^ were acquired. Using the same scan protocol with less acceleration (3.75), three 3D cines (2 × 2 × 2 mm^3^) in breath hold (BH) at an arbitrary stage were acquired within 20 s. The TR-4DMRI images were reconstructed based on the super-resolution approach that has been developed *via* deformable image registration between the low-resolution FB and high-resolution BH images. Detailed 4DMRI scanning and reconstruction methods and conditions can be found in the previous publications (9–11).

The diaphragmatic motion waveforms were extracted from TR-4DMRI using an in-house program in MATLAB (MathWorks, MA). A navigator box (3 × 3 × 6 cm^3^) drawn on the right diaphragmatic dome was used to calculate the average voxel intensity at the same SI positions, and the point with the largest gradient was determined as the diaphragm position. Over the 80 images from a 40-s scan, diaphragmatic motion trajectory in a scan series was used as a motion waveform in the superior–inferior (SI) direction. Each of the three waveforms at the frequency of *f =* 2* Hz* was interpolated using the b-spline function to *f =* 4* Hz* and *f =* 8* Hz* to match the possible scanning rates of clinical 2D cine frame rate, containing a total of 40•*f* timepoints.

### Just-in-Time Prediction to Overcome System Latency in the 2D BEV Cine Strategy

Two time-series forecasting algorithms were applied to predict diaphragm motion based on the motion waveforms: (1) a classical autoregressive (AR) modeling algorithm implemented in the MATLAB Econometric Toolbox™ that uses past values as inputs to a regression algorithm to predict future values and (2) a deep-learning long short-term memory (LSTM) recurrent neural network algorithm in the MATLAB Deep Learning Toolbox™ that processes input data by looping over the time steps and updating the network state containing information over previous time steps. Various parameters were tested for the best prediction accuracy and performance in the two predictive algorithms, and the optimal settings include using 30-s training data, 10 AR polynomial degrees, and 20 hidden layers in the neural network using the Adam (Adaptive Moment Estimation) optimizer with the maximum of 150 epochs.

In each of the interpolated 40-s waveforms, a 30-s waveform section with 30•*f* timepoints was applied as training data to predict the diaphragm position at the next time point in 125 ms at *f* = 8 Hz and 250 ms at *f* = 4 Hz. After a prediction, the training dataset was moved one timepoint forward by adding one new timepoint and removing one old timepoint. The remaining 10-s waveform served as the ground truth to assess the prediction accuracy of n = 10•*f*-1 predictions per motion waveform. For each patient, a total of 3 × (10•*f*-1) predictions were made and evaluated to assess both patient-specific and population-based accuracy of motion prediction.

### Accounting Tumor Motion Hysteresis by Identifying Tumor With Predicted Motion Direction

The 3 × 80 TR-4DMRI images per patient were categorized as in the inhalation and exhalation processes depending on the diaphragm moving direction from the previous timepoint. Therefore, two TR-4DMRI image libraries of inhalation and exhalation were built with roughly 120 images each. The estimated displacement interval on the diaphragm was 30 mm/120 = 0.25 mm, and the exact interval may vary, depending on the motion range, speed, and number of images. Note that the motion interval can be reduced, as more 40-s TR-4DMRI series could be acquired and added from simulation.

When the next diaphragm position is predicted, the moving direction was first used to select the TR-4DMRI library (inhalation or exhalation), and the predicted amplitude was then applied to identify a matched diaphragm, and therefore the corresponding tumor volume in the library. As exact diaphragm matching may not be found in a library, a small uncertainty should be added on top of the prediction uncertainty for the diaphragm position displacement (*∆D*), namely,


(*1)*
$$\Delta D^{matched}=\Delta D^{prediction}+\Delta D^{matching}$$

Once a volumetric image was identified with a matched diaphragm from a library, either for inhalation or for exhalation, the tumor volume projection was evaluated as the difference in the center of mass (*∆*COM) and the DICE similarity index, compared with the image ground truth embedded in the simulation dataset for accuracy assessment.

### Accuracy of Tumor Volume Projection on 2D BEV Cine for MR-Guided IMRT and VMAT

For IMRT treatment, six fixed beam angles (0°–150°) with 30° intervals were used to assess the *∆*COM and DICE of the projected tumor volumes between the predicted and ground truth as a function of time during treatment delivery. Mimicking a 6-beam IMRT plan, all TR-4DMRI images in the patient-specific libraries were segmented for tumor volume using an automated MATLAB program on all cuts parallel to the BEV with a 2-mm interval and ready to be projected to the 2D BEV images. The union of all projected contours was used as the final tumor volume projection.

For VMAT treatment, the full rotation was divided into 240 sections with 1.5° intervals in 4-Hz simulation and used to assess the *∆*COM and DICE between the tumor volume projections from the predicted and ground truth. In the 8-Hz simulation, 480 sections per gantry rotation with 0.75° intervals were used. The results were analyzed as a function of gantry angle, assuming that the gantry rotates at a constant speed. It should be noted that clinically the gantry position is known within the MR-integrated Linac system and the beam angle will be updated. In the VMAT cases, much more beam angles were prepared with pre-segmented tumor volume ready to be used.

For both IMRT and VMAT cases, the results of tumor volume projection resulting from the predictions at 125 and 250 ms were evaluated using the ∆COM and Dice index based on 8- and 4-Hz interpolated waveforms, respectively. The two-tailed Student’s t test was used for all comparison, and a *p*-value of less than 0.05 was considered statistically significant different.

## Results

### Prediction Accuracy of the AR Modeling and LSTM Deep-Learning Network

**Figure 2:**
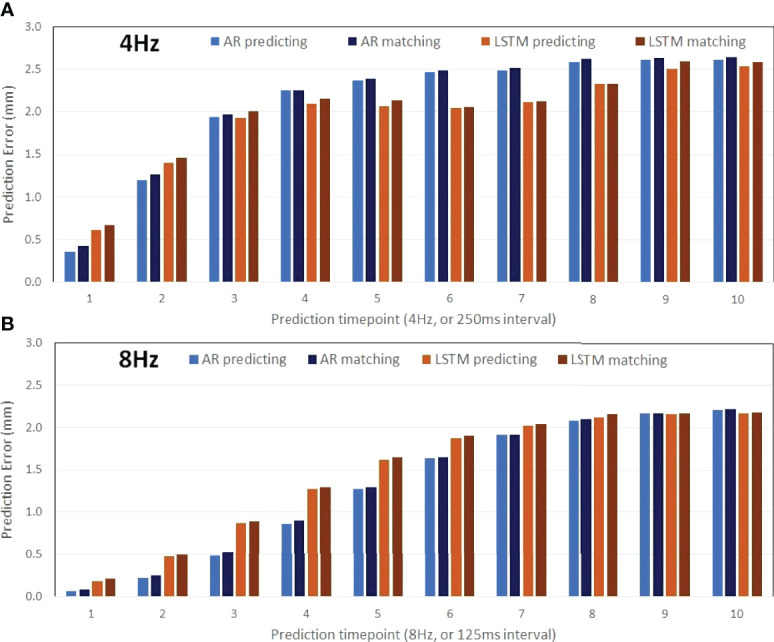
Prediction accuracy of the AR (autoregressive) modeling and the LSTM (long short-term memory) neural network as a function of the time points ahead of training datasets (the 30s of the waveform) using motion waveforms at 4 Hz (**A**: 10 predicted timepoints in 2,500 ms) and at 8 Hz (**B**: 10 predicted timepoints in 1,250 ms). The mean differences increase slightly due to the matching errors.

**Figure 2** illustrates the prediction and matching errors based on the 4- and 8-Hz waveforms. Only the first time point will be used as the diaphragm position in the next 2D BEV cine image for accuracy evaluation, while prediction errors for the first 10 time points are provided, which tend to level off around 1,000 ms at both frequencies. The prediction accuracy is higher at 8 Hz than at 4 Hz, primarily due to more training points being used. Furthermore, the AR method predicts more accurately (0.4 mm) and takes less time (0.4 s) than the LSTM prediction (0.6 mm, 1.5 s) at 4 Hz under the current computing conditions, as shown in **Table 1**. The same trend is observed using the 8-Hz waveform, as shown in **Table 2**.

**Table 1:** Prediction accuracy with matching uncertainties based on 4Hz motion waveforms.

Patient	Diaphragm motion (mm)	4DMRI libraries		AR accuracy (mm)*^b^*					LSTM accuracy (mm)*^b^*				
		Inhale phases	Exhale phases	Prediction			Matched		Prediction			Matched	
				AVG*^a^*	STD	t (s)	AVG*^a^*	STD	AVG*^a^*	STD	t (s)	AVG*^a^*	STD
1	15.8	89	122	0.24	0.20	0.44	0.30	0.20	0.42	0.28	1.47	0.47	0.32
2	12.9	97	118	0.26	0.20	0.44	0.31	0.17	0.59	0.55	1.46	0.62	0.58
3	20.7	108	110	0.44	0.36	0.44	0.47	0.36	0.88	0.70	1.50	0.93	0.70
4	14.4	108	111	0.45	0.32	0.44	0.48	0.35	0.61	0.39	1.49	0.64	0.38
5	9.6	101	117	0.25	0.19	0.44	0.31	0.18	0.24	0.21	1.45	0.27	0.21
6	15.8	114	102	0.33	0.28	0.44	0.47	0.49	0.64	0.61	1.47	0.65	0.63
7	11.6	95	122	0.42	0.24	0.44	0.42	0.26	0.75	0.48	1.45	0.78	0.51
8	29.1	92	125	0.47	0.49	0.44	0.63	0.49	0.81	1.34	1.45	0.97	1.24
AVG	16.2	100.5	115.9	0.36	0.29	0.44	0.42	0.31	0.62	0.57	1.47	0.67	0.57
STD	6.2	8.8	7.7	0.10	0.10	0.00	0.11	0.13	0.21	0.35	0.02	0.23	0.32

The AR (autoregression) and LSTM (long short-term memory) prediction accuracy and performance, diaphragm motion, two TR-4DMRI libraries in the inhalation and exhalation, and the library matching errors are provided. The AR method provides more accurate prediction results using less time than that of the LSTM method.

^a^p-value <0.02 for both prediction and matched accuracy between AR and LSTM.

^b^No significant difference between prediction and matched accuracy using either AR or LSTM.

**Table 2:** Prediction accuracy with matching uncertainties based on 8-Hz motion waveforms.

Patient	Tumor motion and volume				AR accuracy (mm)*^b^*					LSTM accuracy (mm)*^b^*				
	SI (mm)	AP (mm)	LR (mm)	Vol (cc)	Prediction			Matched		Prediction			Matched	
					AVG*^a^*	STD	t (s)	AVG*^a^*	STD	AVG*^a^*	STD	t (s)	AVG*^a^*	STD
1	3.6	6.7	2.1	5.2	0.03	0.03	0.52	0.05	0.06	0.14	0.12	1.87	0.19	0.13
2	9.1	8.0	5.2	16.2	0.05	0.04	0.51	0.06	0.05	0.20	0.17	1.85	0.22	0.16
3	6.6	5.5	8.9	8.8	0.08	0.06	0.50	0.10	0.07	0.15	0.12	1.93	0.19	0.13
4	3.7	4.5	4.1	25.1	0.06	0.05	0.51	0.07	0.06	0.17	0.13	1.89	0.20	0.14
5	3.5	2.1	2.1	3.5	0.04	0.04	0.51	0.06	0.04	0.11	0.09	1.90	0.13	0.10
6	6.0	8.9	9.9	10.0	0.10	0.09	0.50	0.14	0.11	0.17	0.14	1.93	0.20	0.14
7	8.1	8.4	6.5	64.1	0.05	0.04	0.50	0.07	0.05	0.15	0.12	1.92	0.16	0.11
8	5.2	2.7	5.3	1.2	0.06	0.07	0.44	0.09	0.11	0.30	0.45	1.89	0.37	0.51
AVG	5.7	5.9	5.5	16.8	0.06	0.05	0.50	0.08	0.07	0.18	0.17	1.90	0.21	0.18
STD	2.1	2.6	2.9	20.6	0.02	0.02	0.03	0.03	0.03	0.06	0.11	0.03	0.07	0.14

The AR (autoregression) and LSTM (long short-term memory) prediction accuracy and performance, tumor motion, location, and volume, and the library matching errors are provided. The AR method provides more accurate prediction results using less time than that of the LSTM method.

^a^p-value <0.001 in both prediction and matched accuracy between AR and LSTM.

^b^No significant difference between prediction and matched accuracy using either AR or LSTM.

### Tumor Motion Hysteresis and Compensation Using Exhalation and Inhalation Libraries

**Figure 3:**
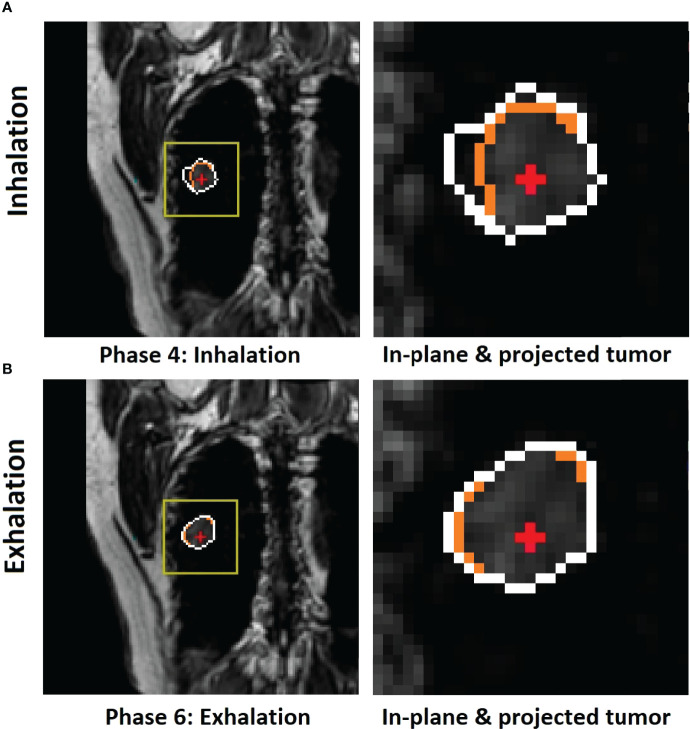
Illustration of motion hysteresis of a posterior peripheral tumor (patient 2, with 20 breathing cycles in 40 s) during mid-inhalation **(A)** and mid-exhalation **(B)** with a similar diaphragm displacement at the zero-gantry angle (BEV = 0°). The isocenter (red cross) position is shown inside the in-plane tumor BEV contour (orange) and the projected volumetric tumor contour (white). Both the in-plane and projected tumor contour shapes and the centers of mass (COMs) are different between inhalation and exhalation. By selectively using either the inhalation or exhalation TR-4DMRI library based on the motion direction, the respiratory hysteresis effect is compensated.

**Figure 4:**
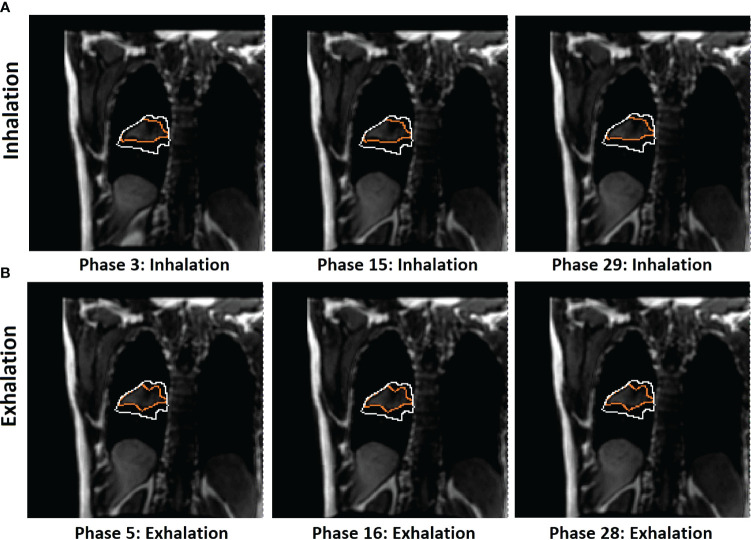
Illustration of 2D BEV (beam angle = 0°) difference of in-plane tumor contours (orange) between mid-inhalation **(A)** and mid-exhalation **(B)** (patient 7, with 17 breathing cycles in 40 s), while projected tumor volume contours (white) are similar in the multi-cycle TR-4DMRI images for this patient. The diaphragm positions are similar in all cases. Due to respiratory hysteresis, including the AP motion, the in-plane tumor contours (orange) are distinctively different between inhalation and exhalation. In contrast, tumor volume projections may not be affected by a through-plane (AP) motion, presenting a stable projected tumor contour. This predictive strategy distinguishes hysteresis-caused tumor volume differences *via* selectively using either the inhalation or exhalation TR-4DMRI library based on the respiratory direction, while the previous method distinguishes them *via* searching for a match with the highest Dice similarity index (8).

The motion of the diaphragm spends slightly more time (more images) in exhalation than inhalation phases, as shown in **Table 1**. **Figures 3**, **4** show a couple of examples of motion hysteresis of the tumors in the coronal view (BEV = 0°) and are compensated for by identifying the matched tumor volume using the appropriate inhalation or exhalation TR-4DMRI library.

### Verification of 2D BEV Cine With Tumor Volume Projections for IMRT and VMAT Treatments

**Figure 5:**
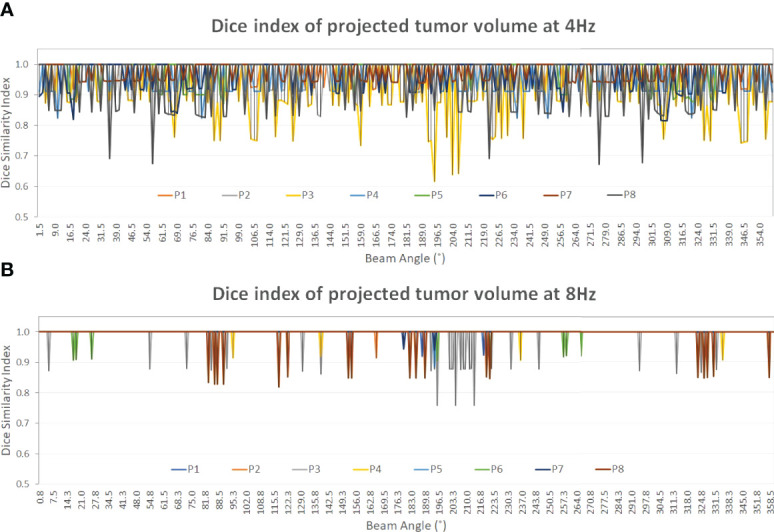
The Dice similarity index of the projected tumor contours on the 2D BEV cine images between the predicted and the ground truth of the tumor volume as a function of beam angle (0°–360°). A constant gantry rotation is assumed for the two plots (Dice value display: 0.4–1.0). At both frequencies, the Dice index of patient 3 (yellow) shows the most variations among the eight patients. From 4 Hz **(A)** to 8 Hz **(B)** the average Dice index increases from 0.97 ± 0.02 to 1.00 ± 0.00 (**Table 3**) as the prediction accuracy increases, as shown in **Tables 1**, **2**.

**Table 3** shows the average and standard deviation of COM difference (∆COM) and Dice similarity of projected tumor volume between the identified and ground truth for IMRT with six fixed beam angles and for VMAT with rotating beam angles (1.5° interval at 4 Hz and 0.75° interval at 8 Hz). **Figure 5** shows the Dice similarity index in VMAT as a function of beam angle for predictions using the 4- and 8-Hz waveform data.

**Table 3:** The accuracy of the center of mass (∆COM) and shape (Dice similarity index) of the projected tumor volume onto the 2D BEV cine images.

Patient	4-Hz motion waveforms^*^						8-Hz motion waveforms^*^					
	IMRT			VMAT			IMRT			VMAT		
	∆COM*^a^* (mm)	2D*^b^* Dice	3D*^c^* Dice	∆COM*^a^* (mm)	2D*^b^* Dice	3D*^c^* Dice	∆COM*^a^* (mm)	2D*^b^* Dice	3D*^c^* Dice	∆COM*^a^* (mm)	2D*^b^* Dice	3D*^c^* Dice
1	0.25	0.98	0.99	0.25	0.98	0.99	0.06	1.00	1.00	0.06	1.00	1.00
2	0.31	0.97	0.98	0.31	0.97	0.98	0.07	1.00	1.00	0.07	1.00	1.00
3	0.64	0.88	0.93	0.64	0.88	0.93	0.17	0.98	0.98	0.17	0.98	0.99
4	0.47	0.94	0.96	0.47	0.94	0.96	0.11	0.99	0.99	0.11	0.99	1.00
5	0.25	0.98	0.99	0.25	0.98	0.99	0.06	0.99	0.99	0.06	0.99	1.00
6	0.42	0.96	0.97	0.42	0.95	0.97	0.12	0.99	0.99	0.12	0.99	1.00
7	0.40	0.97	0.98	0.40	0.97	0.98	0.07	1.00	1.00	0.07	1.00	1.00
8	0.40	0.93	0.95	0.40	0.93	0.95	0.10	0.99	0.99	0.10	0.99	0.99
AVG	0.39	0.95	0.97	0.39	0.95	0.97	0.10	0.99	1.00	0.10	0.99	1.00
STD	0.13	0.03	0.02	0.13	0.03	0.02	0.04	0.01	0.00	0.04	0.01	0.00

At both frequencies of the waveforms for both IMRT and VMAT, a sub-mm accuracy in COM and a greater than 0.95 Dice on average are achieved using the predictive strategy. Additionally, as the prediction accuracy is higher at 8 Hz than at 4 Hz, the accuracy of tumor volume projection is also higher, suggesting the benefit of scanning 2D BEV cine at the highest possible frequency.

^a^p-value <0.0001 for ∆COM comparison between 4- and 8-Hz motion waveform.

^b^2D Dice refers to the 2D tumor contour on the 2D BEV cine image. p-value < 0.005 for 2D Dice comparison between 4- and 8-Hz motion waveform.

^c^3D Dice refers to the tumor volume projection on the 2D BEV cine image. p-value <0.01 for 3D Dice comparison between 4- and 8-Hz motion waveform.

*No significant difference between ∆COM, 2D Dice and 3D Dice in both frequencies of motion waveform.

## Discussion

Based on the previous 2D BEV cine approach for MR-guided IMRT (8), we have demonstrated the following three improvements of the approach in this simulation study: (1) the computation latency has been minimized by applying the prediction method in parallel with the 2D cine scan to identify a motion-matched tumor volume without searching from scratch, (2) respiratory-induced tumor motion hysteresis has been explicitely compensated for using the predicted motion to find a match in either inhalation or exhalation libraries depending on the motion direction, and (3) the feasibility of the 2D BEV cine approach to MR-guided VMAT has been tested. In the following, the advantages and limitations of the new 2D BEV cine approach will be discussed in depth.

### Minimizing Computation Latency Using Just-in-Time Tumor Motion Prediction

The so-called just-in-time tumor motion prediction has been studied to combat the system latency in respiratory-gated or tumor-tracking radiotherapy, as it can predict several tens or hundreds of milliseconds (ms) ahead of time, allowing the system to start to act before an event occurs. In this study, the predictive strategy is applied to remove the computation latency in the 2D–3D library matching (8) for tumor volume projection. As the prediction can be started in parallel with the next BEV 2D cine acquisition, the predicted tumor position in the next cine frame should be available and ready to project tumor volume contour onto the 2D BEV cine image. Therefore, the computation latency can be reduced or eliminated if the prediction can be completed within 125 or 250 ms for 8- or 4-Hz cine acquisition, respectively.

Interestingly, the prediction accuracy and speed of the AR modeling method outperform the deep-learning-based LSTM neural network method, in both accuracy and performance. As the learning process is progressively reoccurring along with the moving training dataset, the LSTM has to relearn every time in the current algorithm. Therefore, the AR results will be used as the focus of the discussion. As shown in **Tables 1**, **2**, the current AR computation times for prediction are 400 and 500 ms for 4- and 8-Hz training datasets, respectively. The computation time must be reduced to within 125–250 ms to remove the computation latency, and performance enhancement is achievable by optimizing the prediction code, using a more powerful computer, and/or employing parallel computing techniques, including the graphics processing unit (GPU) technique.

On average, the prediction accuracy is higher for the time point that is closer to the training dataset, as illustrated by the predictions of the next 125 ms (8 Hz) and 250 ms (4 Hz), as shown in **Tables 1**, **2**. The higher the 2D cine frame rate, the higher the accuracy of prediction would be, and therefore the more accurate the tumor volume projection. Therefore, it is recommended to apply the highest cine frame rate, as long as the cine image quality is sufficient for tumor visualization. In this study, as the b-spline interpolation was applied to the waveform extracted from TR-4DMRI (2Hz), the waveforms are “smoother” than the actual, which contains random noise. However, even if a 2% random error was added to the interpolated motion waveform, the prediction error should still be at the sub-mm level. A random library matching error was present due to the limited motion interval in the TR-4DMRI library, and on average, the prediction results remain roughly the same (**Figure 2**).

### Compensating for the Tumor Motion Hysteresis by Separating Inhalation and Exhalation

It is well known that respiratory-induced tumor motion is direction-dependent, meaning the path for inhalation is different from that of exhalation, namely, motion hysteresis (21, 24). Therefore, when identifying an image with a matching diaphragm or tumor displacement in the SI direction, the motion direction should be considered, in addition to amplitude. In this study, we split the TR-4DMRI library into inhalation and exhalation libraries and applied motion direction first to select the correct library and then found a match with the motion amplitude. Therefore, the hysteresis effect will be accounted for.

Using the current three-series TR-4DMRI, the number of images seems sufficient to have a small-enough motion interval in both inhalation and exhalation libraries, as the uncertainty from slight mismatching does not add too much error on average (**Figure 2**). However, for individual prediction, the finer interval should be helpful to identify a precise diaphragm or tumor position. The library sizes can be easily increased by acquiring more 40-s TR-4DMRI series as additional acquisitions. The 40-s limit is due to the MR scanner memory capacity. Therefore, the number of MR images in the exhalation and inhalation libraries can be increased substantially within a couple of minutes of TR-4DMIR acquisition.

The motion hysteresis has some variation, meaning the inhalation and exhalation path may not be very reproducible, as part of breathing irregularities. Therefore, there is an uncertainty in identifying a tumor volume from the libraries, either through previous 2D–3D library matching (8) or through current inhalation/exhalation library assignment.

### Advantages and Limitations of Using BEV 2D Cine for MRgRT Treatments

It is a commonly accepted concept in image-guided radiotherapy that BEV imaging is the most useful guidance because it directly verifies if the radiation beam is targeting a mobile tumor and if beam-to-tumor conformality is acceptable by comparing the projected tumor volume in the planning BEV image and the verification BEV image. In a conventional Linac, an MV electronic portal imaging device (EPID) is used to take the BEV portal image, while in an MR-integrated Linac, a 2D BEV cine image can be acquired by changing the MR imaging orientation to be perpendicular to the radiation beam. Aside from other differences, the 2D BEV cine image provides a slice image, while the EPID produces a radiographic projection image. Therefore, the through-plane tumor motion will affect the MR cine image, and projecting the tumor volume onto the BEV cine becomes necessary. To retrieve tumor volumetric information, we proposed to identify the matched tumor volume in TR-4DMRI libraries *via* either previous 2D–3D library matching (8) or the predictive strategy to identify the motion-matched and hysteresis-compensated tumor volume for volume projection onto the 2D BEV cine image. Although both approaches work, the current approach has the advantage of no computational latency.

In this study, verification of the predicted tumor volume projection against the ground truth illustrates the accuracy of the improved 2D BEV cine strategy. A sub-mm accuracy in prediction and tumor COM has been achieved, while the contour shape similarity of the projected tumor volume is as high as >0.95 against the ground truth. Therefore, the 2D BEV cine with projected tumor volume is sufficiently accurate and reliable and can be viewed in real time as soon as the 2D BEV cine is acquired without computational latency. The current predictive code needs to be optimized on its performance to reduce the computation time from 400 ms to within 250 ms for 4 Hz, including using parallel computing techniques, such as GPU technology.

In this predictive strategy, a high correlation between the diaphragm and tumor motions was assumed, which is often true for a lung or liver tumor that is located near the diaphragm (25–27). If a tumor is away from the diaphragm, its motion amplitude should be only a fraction of that of the diaphragm. Therefore, the tumor volume can be found with an acceptable tolerance when the diaphragm position has a precise match. As the diaphragm is a large object, uncertainties in its position determination are present (28). The sub-mm COM accuracy results have validated the assumption and method of segmenting the diaphragm used in this study. Clinically, if a tumor volume is sufficiently large, the tumor motion trajectory waveform can be directly measured using the same image processing tool on TR-4DMRI images. Clinically, the tumor trajectory from the 2D BEV cine can be also used as the motion of the day (**Figure 1**). Therefore, the error from the imperfect diaphragm–tumor correlation is eliminated, and more accurate identification and projection of tumor volume can be achieved.

Breathing irregularities have been observed and investigated during radiotherapy treatment in comparison with the motion assessment at the simulation (29, 30). Therefore, the TR-4DMRI library may need more images under some “abnormal” breathing conditions, including slightly deeper breathing. Therefore, the TR-4DMRI library represents more possible variations, ensuring the finding of a motion-matched and hysteresis-compensated tumor volume for projection onto the 2D BEV cine image.

Currently, the 2D BEV cine approach with projected tumor volume has only been tested for motion monitoring of peripheral lung cancer; it remains a challenge for centrally located lung tumor, liver, and pancreatic cancer, for which the image contrast between tumor and surrounding normal tissue may not be as clear, making the automatic tumor delineation difficult, even for MR imaging with high soft-tissue contrast. Therefore, further investigation is needed to assess the feasibility of other disease sites.

## Conclusion

This study has demonstrated an improved 2D BEV cine approach for MR-guided IMRT with minimal computation latency, ability to compensating for respiratory hysteresis, and the feasibility for MR-guided VMAT. The predictive method can achieve sub-mm accuracy to determine the diaphragm position, tumor position, and tumor projection for the next frame of the 2D BEV cine image. The AR algorithm outperforms the LSTM algorithm in the next-frame motion prediction at both 4- and 8-Hz 2D BEV cine frame rates. The potential respiratory hysteresis effect on tumor shape between inhalation and exhalation is accounted for by checking motion direction and using corresponding TR-4DMRI libraries. This approach allows real-time assessment of beam-to-tumor conformality for respiratory gating or tracking during IMRT or VMAT treatments. With further clinical testing, this 2D BEV cine approach has strong potential to serve as optimal personalized imaging guidance in current MR-guided IMRT or future VMAT treatments.

## Data Availability Statement

The raw data supporting the conclusions of this article can be made available by the authors upon request without undue reservation.

## Ethics Statement

The studies involving human participants were reviewed and approved by the MSK Institutional Review Board (IRB). The patients/participants provided their written informed consent to participate in this study.

## Author Contributions

Both authors have made substantial contributions to this work. XN conducted most of the coding and computation. GL proposed the idea, participated in the data acquisition and analysis, and wrote the paper. All authors contributed to the article and approved the submitted version.

## Conflict of Interest

The authors declare that the research was conducted in the absence of any commercial or financial relationships that could be construed as a potential conflict of interest.

## Publisher’s Note

All claims expressed in this article are solely those of the authors and do not necessarily represent those of their affiliated organizations, or those of the publisher, the editors and the reviewers. Any product that may be evaluated in this article, or claim that may be made by its manufacturer, is not guaranteed or endorsed by the publisher.
